# Antihyperglycemic and Anti-Inflammatory Effects of Standardized *Curcuma xanthorrhiza* Roxb. Extract and Its Active Compound Xanthorrhizol in High-Fat Diet-Induced Obese Mice

**DOI:** 10.1155/2014/205915

**Published:** 2014-06-24

**Authors:** Mi-Bo Kim, Changhee Kim, Youngwoo Song, Jae-Kwan Hwang

**Affiliations:** ^1^Department of Biomaterials Science and Engineering, Yonsei University, Seoul 120-749, Republic of Korea; ^2^Department of Biotechnology, College of Life Science and Biotechnology, Yonsei University, Seoul 120-749, Republic of Korea

## Abstract

Xanthorrhizol, a natural compound isolated from *Curcuma xanthorrhiza* Roxb. (Java turmeric), has been reported to possess antioxidant and anticancer properties; however, its effects on metabolic disorders remain unknown. The aim of the present study was to evaluate the effects of xanthorrhizol (XAN) and *C. xanthorrhiza* extract (CXE) with standardized XAN on hyperglycemia and inflammatory markers in high-fat diet- (HFD-) induced obese mice. Treatment with XAN (10 or 25 mg/kg/day) or CXE (50 or 100 mg/kg/day) significantly decreased fasting and postprandial blood glucose levels in HFD-induced obese mice. XAN and CXE treatments also lowered insulin, glucose, free fatty acid (FFA), and triglyceride (TG) levels in serum. Epididymal fat pad and adipocyte size were decreased by high doses of XAN (26.6% and 20.1%) and CXE (25.8% and 22.5%), respectively. XAN and CXE treatment also suppressed the development of fatty liver by decreasing liver fat accumulation. Moreover, XAN and CXE significantly inhibited production of inflammatory cytokines, such as tumor necrosis factor-alpha (TNF-*α*), interleukin-6 (IL-6), interleukin-1*β* (IL-1*β*), and C-reactive protein (CRP) in adipose tissue (27.8–82.7%), liver (43.9–84.7%), and muscle (65.2–92.5%). Overall, these results suggest that XAN and CXE, with their antihyperglycemic and anti-inflammatory activities, might be used as potent antidiabetic agents for the treatment of type 2 diabetes.

## 1. Introduction

The worldwide prevalence of type 2 diabetes is related to increased rates of obesity. Obesity causes an insulin-resistant state in target tissues and is a high risk factor for chronic diseases such as type 2 diabetes [1, 2]. Abnormally high blood glucose levels in type 2 diabetes are caused by relative insulin insufficiency, impaired insulin secretion due to beta cell dysfunction, and impaired glucose tolerance [3, 4]. Type 2 diabetes is characterized by increased fatty acid release from adipose tissue, impaired suppression of glucose output in the liver, and reduced insulin-stimulated glucose uptake in muscle [5, 6].

Insulin resistance accompanied by hyperglycemia and hyperlipidemia plays a crucial role in the development of type 2 diabetes [7]. Insulin-resistant states are considered to be chronic low-grade inflammation states, including increased production of proinflammatory molecules such as tumor necrosis factor-alpha (TNF-*α*), interleukin-6 (IL-6), interleukin-1*β* (IL-1*β*), and C-reactive protein (CRP) in adipose tissue, liver, and muscle [1, 5, 6]. These proinflammatory cytokine levels are elevated in insulin target tissues and blood from obese rodents, and neutralization of inflammatory cytokines improves insulin sensitivity. Activation of these inflammatory cytokines can directly induce insulin resistance and disturb intracellular insulin signaling pathways [3, 8]. Furthermore, these inflammatory factors negatively regulate insulin receptor tyrosine phosphorylation, blunt the insulin-stimulated tyrosine phosphorylation of insulin receptor substrate (IRS-1), and reduce transcription of key targets in the insulin signaling cascade, all of which interrupt the transduction of insulin signaling [3, 9].

**Figure 1:**
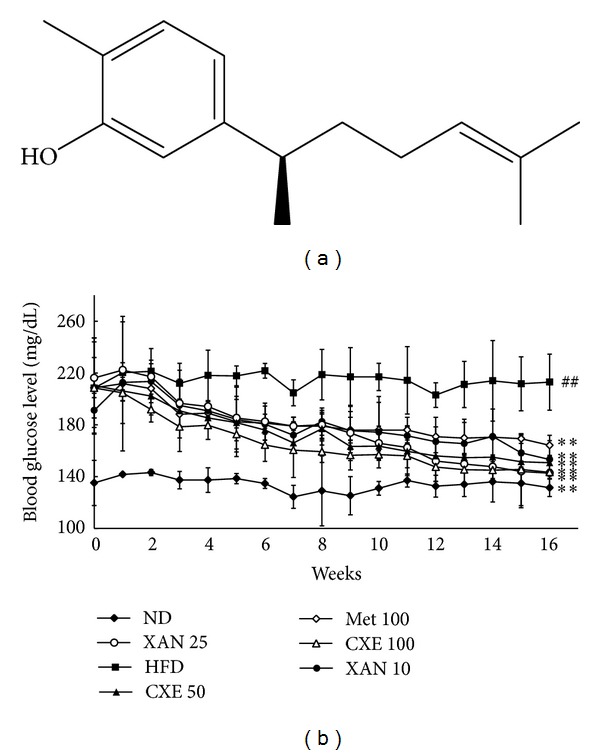
Effects of XAN and CXE treatments on fasting glucose levels in HFD-induced obese mice. (a) Chemical structure of XAN. (b) Changes in fasting blood glucose levels. Mice were administrated normal diet (ND) for 24 weeks. After 8 weeks of HFD-induced obesity, mice were orally administrated with a vehicle HFD (HFD control), HFD with metformin 100 mg/kg/day (Met 100), XAN 10 or 25 mg/kg/day (XAN 10 or 25), or CXE 50 or 100 mg/kg/day (CXE 50 or 100) for 16 weeks. Results are expressed as mean ± SD (*n* = 7). ^##^ *P* < 0.01 compared to the ND group. **P < 0.01 compared to the HFD control group.

*Curcuma xanthorrhiza* Roxb., commonly known as Java turmeric, has been used as a traditional medicinal plant in some tropical countries such as Indonesia and Malaysia for food and medicinal purposes to treat hepatitis, liver disorders, stomach diseases, rheumatism, and skin inflammation [9].* C. xanthorrhiza* contains bioactive compounds, such as curcuminoids, camphor, geranyl acetate, zerumbone, *β*-curcumene, zingiberene, ar-curcumene, and xanthorrhizol [10]. Xanthorrhizol (Figure 1(a)), a sesquiterpenoid compound isolated from the rhizome of* C. xanthorrhiza, *has been reported to possess a variety of biological properties, including antibacterial, antifungal, anticancer, phytoestrogenic, and neuroprotective activities [11–13]; however, its metabolic effects remain to be elucidated. The present study reports the inhibitory effects of* C. xanthorrhiza* extract and its active compound xanthorrhizol on hyperglycemia and proinflammatory markers in high-fat diet- (HFD-) induced obese mice.

## 2. Materials and Methods

### 2.1. Preparation of Standardized* C. xanthorrhiza* Extract

Rhizomes of* C. xanthorrhiza* were collected in Jakarta, Indonesia. A voucher specimen was deposited at the Department of Biotechnology, Yonsei University (Seoul, Korea). The dried rhizomes (100 g) were ground and soaked in 95% ethanol for 2 days at room temperature. The* C. xanthorrhiza* filtrate was concentrated under reduced pressure using a rotary evaporator (Heidolph Instruments GmbH & Co. KG., Schwabach, Germany) to obtain* C. xanthorrhiza *extract (CXE) with a yield of 11.1% (w/w). The standardized CXE contained 16.64% (w/w) of xanthorrhizol as a bioactive compound.

### 2.2. Isolation of Xanthorrhizol

CXE (11.1 g) was separated by silica gel column chromatography (70–230 mesh, Merck & Co., Inc., Whitehouse Station, NJ, USA) eluted with* n*-hexane-ethyl acetate solution (10 : 1, v/v) to give six fractions (fractions I–VI). Fraction V (2.7 g) was further purified by reverse phase (C18) column chromatography (LiChroprep, RP-18, 25–40 *μ*m, Merck & Co., Inc.) eluted with 80% methanol. Compound V-C (0.52 g) was finally obtained as a single compound, xanthorrhizol (XAN). The purity of XAN was determined to be ≥98% by HPLC analysis.

### 2.3. Animal Experiments

Male, 4-week-old C57BL/6 mice (Doo Yeol Biotech, Seoul, Korea) were housed in a controlled environment (25 ± 2°C, 55 ± 5% relative humidity with a 12 h light-dark cycle). After acclimatization for one week, the mice were separately fed a normal diet (ND, Research Diets D12450B, 10% kcal from fat) or HFD (Research Diets D12492, 60% kcal from fat) for 8 weeks. Then, the mice were randomly divided into seven experimental groups (each group, *n* = 7) as follows: group 1 received a ND; group 2 received a HFD; group 3 received the HFD with metformin 100 mg/kg/day (Met 100); group 4 and group 5 received the HFD with XAN 10 and 25 mg/kg/day (XAN 10 or 25), respectively; group 6 and group 7 received the HFD with CXE 50 and 100 mg/kg/day (CXE 50 or 100), respectively. Animals were fed via oral feeding needles for 16 weeks. Throughout the experimental period, body weight and fasting blood glucose were monitored twice per week. Fasting blood glucose was measured from the tail vein of mice using a glucometer (Handok, Seoul, Korea). At the end of the 16-week oral administration period, all mice were sacrificed with diethyl ether after an overnight fast. The fat pads, liver, and muscle tissue were removed, weighed, and frozen on dry ice. This study adhered to the Guide for the Care and Use of Laboratory Animals developed by the Institute of Laboratory Animal Resources of the National Research Council, and it was approved by the Institutional Animal Care and Use Committee (IACUC) of Yonsei University.

### 2.4. Glucose Tolerance Test

Animals were administered with XAN, CXE, and metformin orally for 16 weeks and then challenged with oral glucose (1.5 g/kg body weight) after fasting for 12 hours. Blood samples were obtained from the tail vein immediately at 0, 30, 60, and 120 min after the glucose load. Blood glucose was measured using a glucometer (Handok).

### 2.5. Biochemical Analysis

Blood was collected from mice by heart puncture and held at room temperature for 1 h; serum was then prepared by centrifugation at 4,000 rpm for 15 min and stored at −70°C until analysis. All serum parameters were analyzed by Green Cross Reference Lab (Yongin, Korea). Serum glucose, cholesterol, triglyceride (TG), low-density lipoprotein-cholesterol (LDL-C), and high-density lipoprotein-cholesterol (HDL-C) were analyzed in a Modular Analytics PE (Roche Diagnostics GmbH, Mannheim, Germany) using enzymatic colorimetric test kits (Roche Diagnostics). Serum free fatty acids (FFA) were analyzed in Modular Analytics P (Hitachi High-Technologies Corp. Ltd., Tokyo, Japan) using an enzymatic colorimetric assay based on the acyl-CoA synthetase-acyl-CoA oxidase (ACS-ACOD) method and using a NEEA HR II kit (Wako, Osaka, Japan). Serum insulin was analyzed in VERSAmax tunable microplate reader (Molecular Devices Inc., Sunnyvale, CA, USA) using an Rat/Mouse Insulin enzyme-linked immunosorbent assay kit (Linco Research Inc., St. Charles, MO, USA). Serum levels of hepatotoxicity markers such as serum glutamic oxaloacetic transaminase (SGOT) and serum glutamic pyruvic transaminase (SGPT) were based on the International Federation of Clinical Chemistry (IFCC) method without pyridoxal phosphate (P-5-P) using Modular Analytics PE (Roche Diagnostics).

### 2.6. Histological Analysis

Adipose tissue and liver obtained from the experimental mice were embedded in a tissue-freezing medium (Leica, Wetzlar, Germany) and fixed, as previously described [14]. After fixation, they were stained with hematoxylin and eosin and analyzed for lipid accumulation and adipocyte size with an Eclipse TE2000U Inverted Microscope with twin CCD cameras (magnification, ×200; Nikon, Tokyo, Japan). Adipocyte area and number were quantified using ImageJ software version 1.47 (National Institutes of Health, Bethesda, MD, USA).

### 2.7. Reverse Transcription-Polymerase Chain Reaction (RT-PCR)

Adipose tissues, livers, and muscles were dissected, frozen in liquid nitrogen, and stored at −80°C until use. Total RNA was extracted from adipose tissues, livers, and muscles using Trizol reagent (Invitrogen). The cDNA was converted into a 20 *μ*L reaction containing 2 *μ*g of total RNA, oligo (dT), and Reverse Transcription Premix (ELPIS-Biotech, Daejeon, Korea). PCR amplification of the cDNA products (3 *μ*L) was performed with PCR premix (ELPIS-Biotech, Daejeon, Korea) and the following primer pairs (Bioneer, Daejeon, Korea): TNF-*α* forward 5′-GCG GAG TCC GGG CAG GTC TA-3′ and TNF-*α* reverse 5′-GGG GGC TGG CTC TGT GAG GA-3′ (458 bp); IL-6 forward 5′-CCG GAG AGG AGA CTT CAC AG-3′ and IL-6 reverse 5′-TCC ACG ATT TCC CAG AGA AC-3′ (102 bp); IL-1*β* forward 5′-CCT CAG TGC GGG CTA TGA CC-3′ and IL-1*β* reverse 5′-TCG GCC AAG ACA GGT CGC TC-3′ (213 bp); CRP forward 5′-GAA CTG GCG GGC ACT GAA CTA-3′ and CRP reverse 5′-GGA GGT GCT TCA GGG TTC ACA-3′ (96 bp); *β*-actin forward 5′-CCA CAC CTT CTA CAA TGA GC-3′ and *β*-actin reverse 5′-TGA GGT AGT CAG TCA GGT C-3′ (308 bp). Primers were designed using Primer-BLAST (http://www.ncbi.nlm.nih.gov/tools/primer-blast/). All primers were denatured at 94°C for 5 min prior to PCR amplification. Amplification consisted of 28–32 cycles as follows: denaturing at 94°C for 30 sec, annealing at 56°C for 1 min, and extending at 72°C for 1 min, followed by a final 5 min extending phase at 72°C. PCR was performed using a Gene Amp PCR System 2700 (Applied Biosystems, Foster City, CA, USA). PCR products were electrophoresed by 1.5% agarose gel electrophoresis and visualized using a G:BOX EF imaging system (Syngene, Cambridge, UK) and the Gensnap program. *β*-Actin was used as an internal control.

### 2.8. Statistical Analysis

Data were expressed as mean ± standard deviation (SD) of seven mice in each group. Statistical analysis was performed using SPSS 12.0 (SPSS Inc., Chicago, IL, USA). Group differences were assessed by one-way analysis of variance (ANOVA) followed by Duncan's test. ^##^ *P* < 0.01, **P < 0.01, and *P < 0.05 were considered to be statistically significant.

## 3. Results

### 3.1. XAN and CXE Treatments Attenuate HFD-Induced Fasting Blood Glucose Levels

To determine whether XAN and CXE improve hyperglycemia in an animal model, C57BL/6J mice with HFD-induced obesity received oral doses of XAN (10 or 25 mg/kg/day) or CXE (50 or 100 mg/kg/day) for 16 weeks (Figure 1(b)). The fasting blood glucose levels of the HFD control group were significantly higher than those of the ND group at the end of the study. The fasting blood glucose levels of the XAN 10 and 25 treatment groups decreased by 21.8% and 33.0%, respectively, as compared to the HFD control group, and fasting blood glucose levels of the CXE 50 and 100 groups decreased by 28.5% and 31.2%, respectively. Thus, XAN and CXE regulated the circulating glucose levels in blood and attenuated hyperglycemia in HFD-induced obese mice.

### 3.2. XAN and CXE Treatments Improve HFD-Induced Insulin Sensitivity

**Figure 2:**
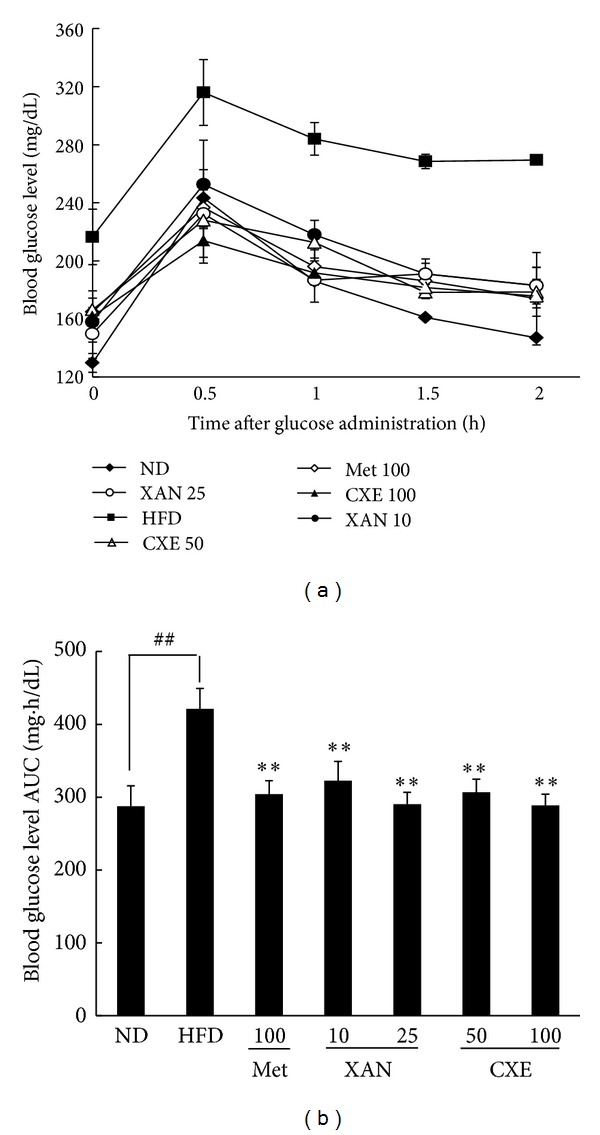
Effects of XAN and CXE treatments on postprandial blood glucose levels in HFD-induced obese mice. (a) At the end of the experiment, the oral glucose tolerance test was performed. (b) The total incremental area under the curve (AUC) for blood glucose was calculated. Results are expressed as mean ± SD (*n* = 7). ^##^ *P* < 0.01 compared to the ND group. **P < 0.01 compared to the HFD control group.

Obesity and being overweight can cause postprandial hyperglycemia, which is important in regulating tight control of blood glucose levels [14]. After oral administration of 25% glucose solution, the blood glucose levels of the HFD control group at 0 min were higher than those of the ND group. The highest blood glucose level was observed at 30 min and decreased in a time-dependent fashion. The XAN and CXE treatment groups showed reduced postchallenge blood glucose levels at all times points compared to the HFD control group (Figure 2(a)). The area under the curve (AUC) analysis showed that the XAN 25 and CXE 100 treatment groups had decreased blood glucose levels of 31.1% and 31.4%, respectively, compared to the HFD control group (Figure 2(b)). These results indicate that XAN and CXE treatments significantly improve insulin resistance.

### 3.3. XAN and CXE Treatments Improve HFD-Induced Epididymal Fat Pad Weight without Reducing Body Weight

**Figure 3:**
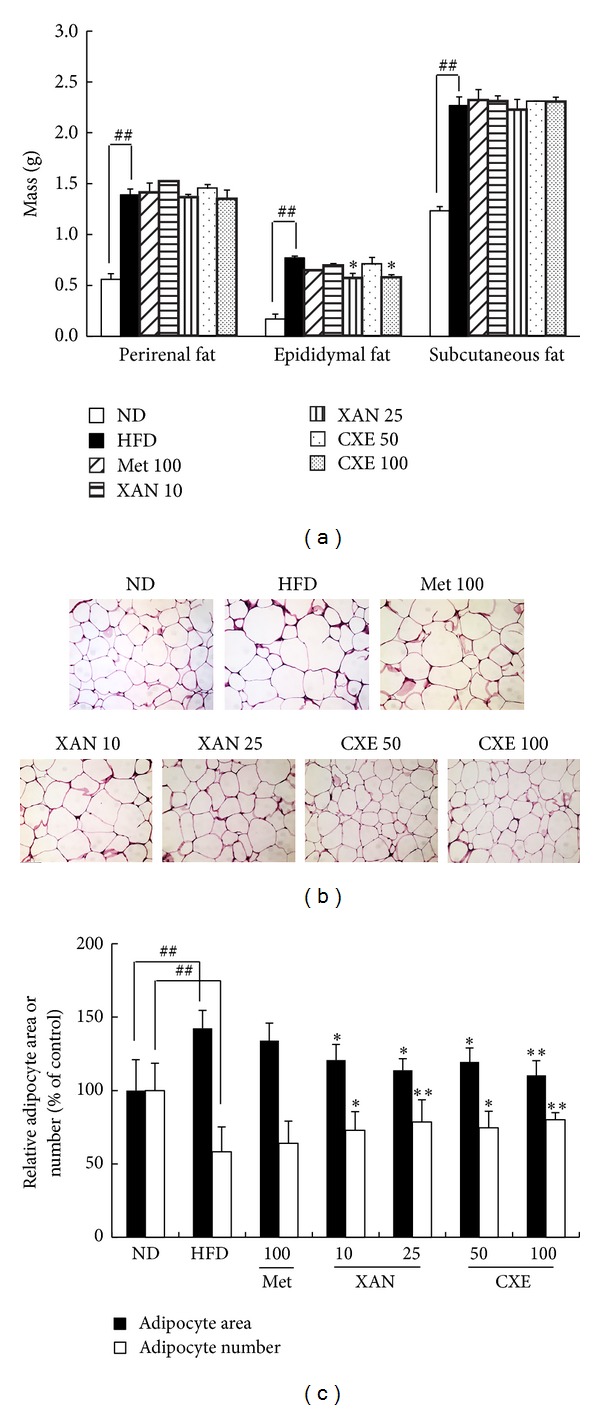
Effects of XAN and CXE treatments on fat pad mass and histological analysis of adipose tissue in HFD-induced obese mice. (a) Fat fad mass in mice fed HFD, XAN, CXE, and metformin. (b) Histological analysis of adipose tissue in mice fed HFD, XAN, CXE, and metformin. (c) Adipocyte area and number in mice fed HFD, XAN, CXE, and metformin. Results are expressed as mean ± SD (*n* = 7). ^##^ *P* < 0.01 compared to the ND group. **P < 0.01 compared to the HFD control group.

The body weights of XAN and CXE treatment groups were not significantly different from that of the HFD control group during the study period (Table 1). However, XAN 25 and CXE 100 treatment groups had significantly reduced epididymal fat pads, reduced by 26.6% and 25.8%, respectively, as compared to the HFD control group (Figure 3(a)). Histological analysis of the epididymal fat indicated that fat mass in the XAN and CXE treatment groups was reduced due to a decrease in adipocyte size and an increase in adipocyte number (Figures 3(b) and 3(c)).

### 3.4. XAN and CXE Treatments Reduce HFD-Induced Insulin, Glucose, FFA, and TG Levels in Serum

High-fat intake generally increases TG and FFA, which can result in insulin resistance by impaired insulin secretion and glucose production [15]. The XAN and CXE treatment groups showed significant reductions in insulin, glucose, FFA, and TG levels compared to the HFD control group (Table 2). The XAN 25 and CXE 100 treatment groups had significantly diminished insulin (34.7% and 44.9%), glucose (33.3% and 30.0%), and FFA (39.9% and 42.0%) levels, respectively, compared to the HFD control group. Moreover, the XAN 10 and CXE 50 treatment groups also showed significantly reduced TG levels (24.7% and 21.1%, resp.) compared to the HFD control group. However, total cholesterol, HDL-C, and LDL-C levels in the serum of XAN and CXE treatment groups were not significantly different from those in the HFD control mice (data not shown). XAN and CXE suppressed obesity-induced hyperlipidemia by decreasing lipid levels in the serum.

### 3.5. XAN and CXE Treatments Suppress HFD-Induced Liver Fat Accumulation and Hepatotoxicity Markers

**Figure 4:**
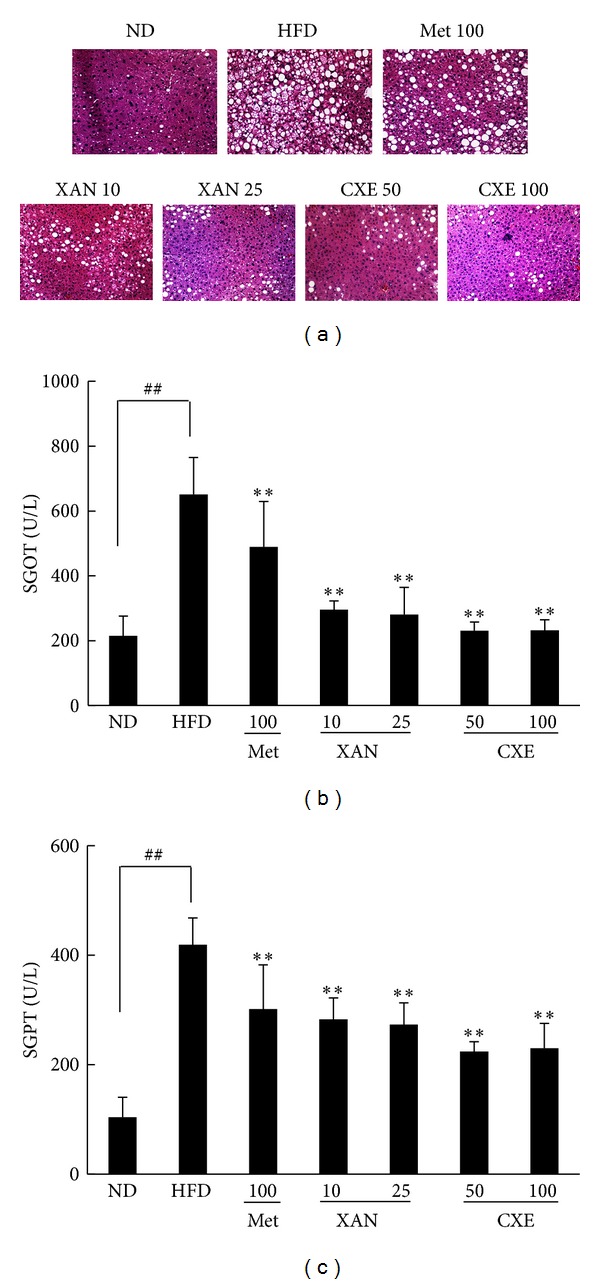
Effects of XAN and CXE treatments on histological analysis of liver tissue and hepatotoxicity markers of serum in HFD-induced obese mice. (a) Histological analysis in liver tissue in mice fed HFD, XAN, CXE, and metformin. (b) The levels of serum glutamic oxaloacetic transaminase (SGOT) and serum glutamic pyruvic transaminase (SGPT). Results are expressed as mean ± SD (*n* = 7). ^##^ *P* < 0.01 compared to the ND group. **P < 0.01 compared to the HFD control group.

The development of fatty liver disease is associated with obesity, insulin resistance, and metabolic syndrome [16]. Histological analysis of the liver tissue from the HFD control group revealed accumulation of fat droplets in the liver, indicating a fatty liver (Figure 4(a)). However, livers from the XAN and CXE treatment groups showed decreased fat droplet size and number, suggesting that XAN and CXE decreased liver fat accumulation and attenuated the fatty liver state in HFD-induced obese mice. To evaluate the effects of XAN and CXE on hepatic function, SGOT and SGPT levels were investigated in HFD-induced obese mice (Figures 4(b) and 4(c)). SGOT and SGPT levels in the HFD control group were significantly higher than those of the ND group, while the XAN and CXE treatment groups showed reduced SGOT and SGPT levels up to the values of the ND group.

### 3.6. XAN and CXE Treatments Decrease Inflammatory Gene Expression in Adipose Tissue

**Figure 5:**
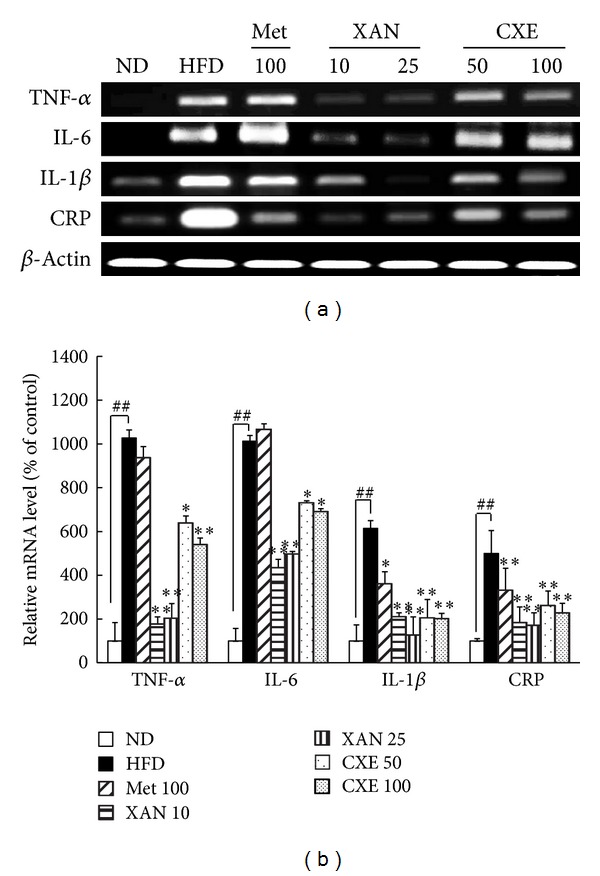
Effects of XAN and CXE treatments on the expression of inflammatory cytokine genes in adipose tissue of HFD-induced obese mice. The expression of TNF-*α*, IL-6, IL-1*β*, and CRP genes in adipose tissue was evaluated by RT-PCR. *β*-Actin was used as an internal control. Results are expressed as mean ± SD (*n* = 7). ^##^ *P* < 0.01 compared to the ND group. *P < 0.05 and **P < 0.01 compared to the HFD control group.

Adipose tissue expansion induces a chronic inflammation state, resulting in the development of insulin resistance and high blood glucose levels [17]. The HFD control group showed upregulation of the TNF-*α*, IL-6, IL-1*β*, and CRP genes (Figure 5). The XAN and CXE treatment groups showed significantly inhibited production of inflammatory cytokines in adipose tissue compared to the HFD control group (Figure 5). The expression of major proinflammatory cytokine, TNF-*α*, was greatly inhibited in the XAN 10 and 25 treatment groups by 82.7% and 80.2%, respectively, compared to the HFD control group. The results suggest that XAN and CXE prevent the recruitment of immune cells to adipose tissue through downregulation of inflammatory cytokine genes.

### 3.7. XAN and CXE Treatments Decrease Inflammatory Gene Expression in Liver

**Figure 6:**
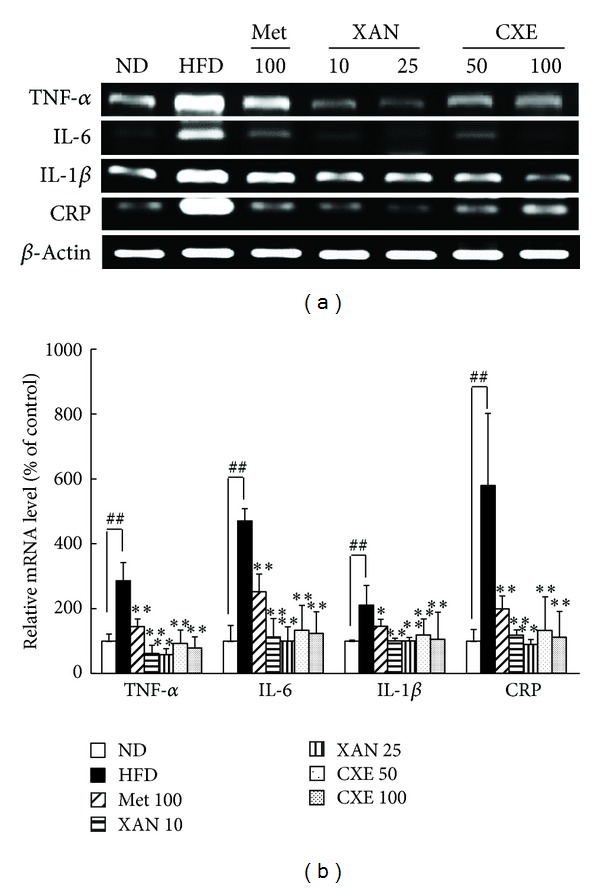
Effects of XAN and CXE treatments on the expression of inflammatory cytokine genes in liver tissue of HFD-induced obese mice. The expression of TNF-*α*, IL-6, IL-1*β*, and CRP genes in liver tissue was evaluated by RT-PCR. *β*-Actin was used as an internal control. Results are expressed as mean ± SD (*n* = 7). ^##^ *P* < 0.01 compared to the ND group. *P < 0.05 and **P < 0.01 compared to the HFD control group.

Inflammatory cytokine secretion in the liver is caused by increasing hepatic NF-*κ*B activity [16]. Inflammatory cytokine levels increased by a HFD were diminished by XAN and CXE treatments in the liver (Figure 6). The nonspecific marker of systemic inflammation, CRP, was inhibited in the XAN 25 and CXE 100 treatment groups by 84.7% and 80.8%, respectively, compared to the HFD control group.

### 3.8. XAN and CXE Treatments Decrease Inflammatory Gene Expression in Muscle

**Figure 7:**
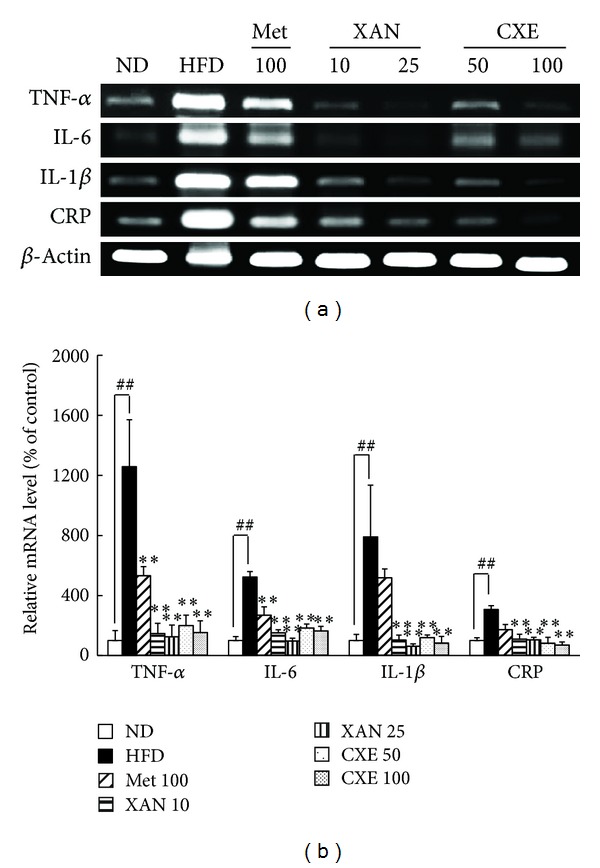
Effects of XAN and CXE treatments on the expression of inflammatory cytokine genes in muscle tissue of HFD-induced obese mice. The expression of TNF-*α*, IL-6, IL-1*β*, and CRP genes in muscle tissue was evaluated by RT-PCR. *β*-Actin was used as an internal control. Results are expressed as mean ± SD (*n* = 7). ^##^ *P* < 0.01 compared to the ND group. *P < 0.05 and **P < 0.01 compared to the HFD control group.

Inflammatory cytokine stimulation prevents insulin signaling in muscle [6]. The HDF-induced inflammatory cytokine levels were significantly reduced by XAN and CXE treatments in muscle (Figure 7). The XAN 25 and CXE 100 treatment groups showed reduced IL-1*β* gene expression by 92.5% and 89.9%, respectively, as compared to the HFD control group.

## 4. Discussion

According to type 2 diabetic therapy, control of fasting and postprandial hyperglycemia is important in maintaining tight control of blood glucose levels. Hyperglycemia leads to insulin resistance and beta cell damage by several different mechanisms, collectively referred to as glucotoxicity [14]. This study demonstrated that XAN and CXE treatments effectively prevented HFD-induced hyperglycemia and insulin resistance by analyzing fasting blood glucose and postprandial blood glucose levels in HFD-induced obese mice (Figures 1 and 2). Furthermore, the antihyperglycemic activities of XAN and CXE were higher than or similar to those of metformin. Metformin has been reported as the first-line oral antihyperglycemic agent for the treatment of type 2 diabetes patients [18]. The findings of the current study suggest that XAN and CXE have protective effects against the development of obesity-induced insulin resistance and high blood glucose levels and could provide a suitable therapeutic approach to the treatment of type 2 diabetes mellitus.

The evaluation of blood glucose and insulin in HFD-induced obese mice is a strong indicator of obesity-induced type-2 diabetes [19]. XAN and CXE treatments significantly decreased insulin and glucose levels in the serum of HFD-induced obese mice (Table 2). Insulin is a hormone that plays an important role in the regulation of blood glucose levels and lipid metabolism in adipose tissue, liver, and muscle [20]. In addition, insulin secretion from pancreatic cells and improvement of glucose metabolism can reduce hyperglycemia [21]. These results suggest that XAN and CXE improve the ability of insulin to stimulate glucose uptake and increase glucose production in HFD-induced obese mice.

HFD increases cellular FFAs and decreases beta-oxidation, ultimately resulting in TG accumulation in serum [22]. Chronic elevation of FFA causes an increase in intracellular TG levels, inhibits the insulin-induced suppression of glycogenolysis and gluconeogenesis, and is strongly associated with reduced hepatic glucose production and improved muscle insulin sensitivity [23]. XAN and CXE reduced the secretion of TG and liberation of FFA in serum, resulting in an attenuation of HFD-induced hyperglycemia (Table 2). Accordingly, XAN and CXE reduced the chronic elevation of FFA and TG in serum, which ameliorated HFD-induced hyperglycemia and insulin resistance.

Excessive adipose tissue is associated with increased fat accumulation and adipose cell hypertrophy, which increases glucose tolerance, fasting glucose levels, and lipid profiles [22]. Furthermore, epididymal fat refers to intraperitoneal fat, which activates FFA and several bioactive adipokines including adiponectin, TNF-*α*, IL-1*β*, and IL-6 [24]. XAN and CXE decreased epididymal fat pad mass and adipocyte size without reducing body weight (Figure 3 and Table 1). These results suggest that XAN and CXE prevent HFD-induced excessive adipose tissue, which leads to attenuating insulin resistance and chronic low-grade inflammation state.

Higher hepatic fat accumulation leads to increased SGPT and SGOT values, which lead to the development of fatty liver disease and hepatic inflammation due to the dysfunction of insulin target cells and resulting imbalance of lipid metabolism [24, 25]. In the present study, XAN and CXE attenuated fatty liver and protected against obesity-induced hepatic injury (Figure 4). Thus, XAN and CXE may regulate an imbalance in hepatic lipid metabolism, glucose production, and inflammation in HFD-induced obese mice.

Insulin resistance is a state of chronic low-grade inflammation, which increases production of inflammatory cytokines, acute-phase reactants, and activates a network of inflammatory signaling pathways [1, 5]. Inflammatory cytokines secreted by adipose tissue have the ability to induce intercellular communication between insulin target cells, including those in adipose tissue, liver, and muscle, which contribute to systemic insulin resistance and an inflammatory state [3, 14]. XAN and CXE markedly reduced inflammation cytokine expression in insulin target tissues, including adipose tissue, liver, and muscle (Figures 5, 6, and 7). Several studies suggest that TNF-*α* and IL-6 improve lipolysis and the secretion of FFA, which promotes insulin resistance by interfering with glucose transport and insulin action [24, 26]. Hyperglycemia induces the production of IL-1*β*, which interferes with insulin signaling and has cytotoxic effects on beta cells, leading to impaired insulin secretion [27]. High CRP levels lead to an increase in NF-*κ*B nuclear translocation, which results in the activation of inflammatory proteins. Therefore, XAN and CXE can attenuate systemic inflammation and the insulin resistance state of obesity-induced hyperglycemia.

CXE, containing 16.64% of XAN, did not show substantial differences as compared to XAN. This may be because CXE contained other bioactive compounds, such as curcuminoids, camphor, geranyl acetate, zerumbone, *β*-curcumene, zingiberene, and ar-curcumene as well as xanthorrhizol [10]. Further studies are necessary to find new antihyperglycemic compounds in CXE.

## 5. Conclusion

XAN and CXE treatments significantly attenuated the HFD-induced hyperglycemic, insulin-resistant, and chronic low-grade systemic inflammatory states in obese mice. XAN and CXE treatments effectively improved serum levels of insulin, glucose, FFA, and TG. In addition, treatment with XAN and CXE decreased epididymal fat pad mass by reducing adipocyte size and liver fat accumulation. It is anticipated that XAN and CXE can be used as natural antidiabetic agents for the prevention of type 2 diabetes mellitus.

## Figures and Tables

**Table 1:** Effects of XAN and CXE treatments on body weight in HFD-induced obese mice.

Group	Initial body weight (g)	Final body weight (g)
ND	30.9 ± 2.3^##^	40.6 ± 2.1^##^
HFD	44.6 ± 3.3	52.0 ± 2.3
Met 100	45.0 ± 2.3	52.5 ± 2.2
XAN 10	44.4 ± 2.4	52.6 ± 3.2
XAN 25	43.9 ± 2.2	50.5 ± 1.6
CXE 50	44.5 ± 3.2	52.3 ± 2.1
CXE 100	45.4 ± 3.4	51.2 ± 1.5

Data are expressed as mean ± SD (*n* = 7). ^##^ *P* < 0.01 compared to the ND group. ND: normal diet, HFD: high-fat diet, Met 100: HFD with metformin 100 mg/kg/day, XAN 10 or 25: HFD with XAN 10 or 25 mg/kg/day, and CXE 50 or 100: HFD with CXE 50 or 100 mg/kg/day.

**Table 2:** Effects of XAN and CXE treatments on serum levels of insulin, glucose, FFA, and TG in HFD-induced obese mice.

Group	Insulin (ng/mL)	Glucose (mg/dL)	FFA (*μ*Eq/L)	TG (mg/dL)
ND	0.9 ± 0.5	208.0 ± 68.7	1170.8 ± 171.9	69 ± 9.9
HFD	4.9 ± 1.0^##^	359.3 ± 36.1^##^	1580.0 ± 152.0^##^	106 ± 30.9^##^
Met 100	4.1 ± 0.9	297.3 ± 50.3	1374.0 ± 166.2	90 ± 14.2
XAN 10	3.5 ± 0.6∗	251.4 ± 8.4∗	1178.3 ± 37.1∗∗	84 ± 8.2∗
XAN 25	3.2 ± 0.6∗	249.5 ± 16.3∗	1054.5 ± 20.5∗∗	87 ± 13.4
CXE 50	3.1 ± 0.7∗	216.0 ± 60.2∗∗	1125.0 ± 120.0∗∗	83 ± 3.1∗
CXE 100	2.7 ± 0.2∗∗	208.3 ± 41.9∗∗	1106.0 ± 67.9∗∗	80 ± 7.6∗

Data are expressed as mean ± SD (*n* = 7). ^##^ *P* < 0.01 compared to the ND group. *P < 0.05 and **P < 0.01 compared to the HFD control group. ND: normal diet, HFD: high-fat diet, Met 100: HFD with metformin 100 mg/kg/day, XAN 10 or 25: HFD with XAN 10 or 25 mg/kg/day, and CXE 50 or 100: HFD with CXE 50 or 100 mg/kg/day.

## References

[1] Arçari DP, Bartchewsky W, dos Santos TW (2011). Anti-inflammatory effects of *yerba maté* extract (*Ilex paraguariensis*) ameliorate insulin resistance in mice with high fat diet-induced obesity. *Molecular and Cellular Endocrinology*.

[2] Vazquez-Prieto MA, Bettaieb A, Haj FG, Fraga CG, Oteiza PI (2012). (-)-Epicatechin prevents TNF*α*-induced activation of signaling cascades involved in inflammation and insulin sensitivity in 3T3-L1 adipocytes. *Archives of Biochemistry and Biophysics*.

[3] Olefsky JM, Glass CK (2009). Macrophages, inflammation, and insulin resistance. *Annual Review of Physiology*.

[4] Taylor R (1999). The nature of type 2 diabetes: the role for new agents. *International Journal of Clinical Practice*.

[5] van Greevenbroek MMJ, Schalkwijk CG, Stehouwer CDA (2013). Obesity-associated low-grade inflammation in type 2 diabetes mellitus: causes and consequences. *Netherlands Journal of Medicine*.

[6] Shoelson SE, Lee J, Goldfine AB (2006). Inflammation and insulin resistance. *Journal of Clinical Investigation*.

[7] Pravenec M, Kazdová L, Cahová M (2006). Fat-specific transgenic expression of resistin in the spontaneously hypertensive rat impairs fatty acid re-esterification. *International Journal of Obesity*.

[8] Snel M, van Diepen JA, Stijnen T (2011). Immediate and long-term effects of addition of exercise to a 16-week very low calorie diet on low-grade inflammation in obese, insulin-dependent type 2 diabetic patients. *Food and Chemical Toxicology*.

[9] Musfiroh I, Muchtaridi M, Muhtadi A (2013). Cytotoxicity studies of xanthorrhizol and its mechanism using molecular docking simulation and pharmacophore modelling. *Journal of Applied Pharmaceutical Science*.

[10] Jantan I, Saputri FC, Qaisar MN, Buang F (2012). Correlation between chemical composition of *Curcuma domestica* and *Curcuma xanthorrhiza* and their antioxidant effect on human low-density lipoprotein oxidation. *Evidence-Based Complementary and Alternative Medicine*.

[11] Hwang J-K, Shim J-S, Baek N-I, Pyun Y-R (2000). Xanthorrhizol: a potential antibacterial agent from *Curcuma xanthorrhiza* against *Streptococcus mutans*. *Planta Medica*.

[12] Choi M-A, Kim SH, Chung W-Y, Hwang J-K, Park K-K (2004). Xanthorrhizol, a natural sesquiterpenoid from *Curcuma xanthorrhiza*, has an anti-metastatic potential in experimental mouse lung metastasis model. *Biochemical and Biophysical Research Communications*.

[13] Lim CS, Jin D-Q, Mok H (2005). Antioxidant and antiinflammatory activities of xanthorrhizol in hippocampal neurons and primary cultured microglia. *Journal of Neuroscience Research*.

[14] Luquet S, Gaudel C, Holst D (2005). Roles of PPAR delta in lipid absorption and metabolism: a new target for the treatment of type 2 diabetes. *Biochimica et Biophysica Acta*.

[15] Hussain SA, Ahmed ZA, Mahwi TO, Aziz TA (2012). Quercetin dampens postprandial hyperglycemia in type 2 diabetic patients challenged with carbohydrates load. *International Journal of Diabetes Research*.

[16] Jang E-M, Choi M-S, Jung UJ (2008). Beneficial effects of curcumin on hyperlipidemia and insulin resistance in high-fat-fed hamsters. *Metabolism: Clinical and Experimental*.

[17] Gaggini M, Morelli M, Buzzigoli E, DeFronzo RA, Bugianesi E, Gastaldelli A (2013). Non-alcoholic fatty liver disease (NAFLD) and its connection with insulin resistance, dyslipidemia, atherosclerosis and coronary heart disease. *Nutrients*.

[18] Tateya S, Tamori Y, Kawaguchi T, Kanda H, Kasuga M (2010). An increase in the circulating concentration of monocyte chemoattractant protein-1 elicits systemic insulin resistance irrespective of adipose tissue inflammation in mice. *Endocrinology*.

[19] Lacroix IM, Li-Chan EC (2013). Overview of food products and dietary constituents with antidiabetic properties and their putative mechanisms of action: a natural approach to complement pharmacotherapy in the management of diabetes. *Molecular Nutrition & Food Research*.

[20] Jung JY, Lim Y, Moon MS, Kim JY, Kwon O (2011). Onion peel extracts ameliorate hyperglycemia and insulin resistance in high fat diet/streptozotocin-induced diabetic rats. *Nutrition and Metabolism*.

[21] Davaatseren M, Hur HJ, Yang HJ (2013). Taraxacum official (dandelion) leaf extract alleviates high-fat diet-induced nonalcoholic fatty liver. *Food and Chemical Toxicology*.

[22] Bansal P, Paul P, Mudgal J (2012). Antidiabetic, antihyperlipidemic and antioxidant effects of the flavonoid rich fraction of *Pilea microphylla* (L.) in high fat diet/streptozotocin-induced diabetes in mice. *Experimental and Toxicologic Pathology*.

[23] Koenen TB, Stienstra R, Van Tits LJ (2011). Hyperglycemia activates caspase-1 and TXNIP-mediated IL-1*β* transcription in human adipose tissue. *Diabetes*.

[24] del Prato S (2009). Role of glucotoxicity and lipotoxicity in the pathophysiology of Type 2 diabetes mellitus and emerging treatment strategies. *Diabetic Medicine*.

[25] Gustafson B (2010). Adipose tissue, inflammation and atherosclerosis. *Journal of Atherosclerosis and Thrombosis*.

[26] Chang Y-Y, Yang D-J, Chiu C-H, Lin Y-L, Chen J-W, Chen Y-C (2013). Antioxidative and anti-inflammatory effects of polyphenol-rich litchi (*Litchi chinensis* Sonn.)-flower-water-extract on livers of high-fat-diet fed hamsters. *Journal of Functional Foods*.

[27] Moloney F, Toomey S, Noone E (2007). Antidiabetic effects of *cis*-9, *trans*-11-conjugated linoleic acid may be mediated via anti-inflammatory effects in white adipose tissue. *Diabetes*.

